# Juvenile Lesions of the Cerebellar Fastigial Nucleus Cause Lasting Cognitive Deficits and Prefrontal Cortex Dysfunction in Adult Rats: Implications for the Cerebellar Cognitive Affective Syndrome

**DOI:** 10.3390/brainsci15080862

**Published:** 2025-08-13

**Authors:** Franziska Maria Decker, Jonas Jelinek, Franck Fogaing Kamgaing, Mesbah Alam, Shadi Al-Afif, Joachim K. Krauss, Kerstin Schwabe, Elvis J. Hermann

**Affiliations:** 1 Department of Neurosurgery, Hannover Medical School, 30625 Hannover, Germanyschwabe.kerstin@mh-hannover.de (K.S.);; 2 Center for Systems Neuroscience (ZSN), 30559 Hannover, Germany

**Keywords:** cerebellar cognitive affective syndrome, auditory oddball paradigm, event-related potentials, rats, prefrontal cortex

## Abstract

**Background/Objectives:** Cerebellar cognitive affective syndrome (CCAS) is a well-recognized postoperative complication in children following resection of brain tumors involving cerebellar midline structures. The fastigial nucleus is regarded as relevant, but the underlying neural mechanisms remain incompletely understood. This study uses an oddball paradigm designed to model attentional and learning processes relevant to CCAS to investigate how early-life lesions of the fastigial nucleus in rats affect cognitive performance and neural information processing in the medial prefrontal cortex (mPFC) in adulthood. **Methods:** Fastigial lesions were induced stereotaxically in 23-day-old male Sprague Dawley rats [*n* = 9]. Naïve [*n* = 9] and sham-lesioned rats [*n* = 6] served as controls. As adults, all rats were trained in an oddball paradigm requiring discrimination of a rare target tone from a rare distractor and a frequent standard tone. Local field potentials (LFPs) were recorded from electrodes implanted in the mPFC during oddball testing and event-related potentials (ERPs) were analyzed. **Results:** Rats with fastigial lesions required significantly more training days to reach ≥70% correct performance criterion. In fully trained rats, analysis of neural recordings during behavioral testing revealed reduced ERP amplitudes and prolonged latencies of late ERP components after target stimuli. Developmental fastigial lesions lead to lasting deficits in cognitive learning capacity and neural mPFC processing, highlighting the integrative role of cerebellar midline structures in higher-order cognitive function and sensory discrimination. **Conclusions:** This rodent model provides a valuable translational platform for further investigating the neural basis of CCAS and may inform neurosurgical strategies aimed at minimizing cognitive sequelae in children undergoing cerebellar tumor resection.

## 1. Introduction

Cerebellar mutism syndrome (CMS) is a little-understood complication following the surgical resection of pediatric brain tumors in the posterior cranial fossa [1]. It is defined as a “silent” period with preserved vigilance, lack of drive and inability to appropriately react to social stimuli [2]. Although the core symptoms of CMS are typically transient, a considerable proportion of affected children exhibit long-term social and cognitive deficits with consequences for their quality of life, known as cerebellar cognitive affective syndrome (CCAS) [3,4,5]. In this context, the term “posterior fossa syndrome” is used in the clinical literature to describe this spectrum of postoperative symptoms, encompassing both the acute phase referred to as CMS and the longer-term impairments summarized under CCAS [2]. Advances in operative neurosurgical techniques and interdisciplinary oncological treatments have significantly improved survival rates and even allow full recovery from tumors [6,7]. The anatomical correlate associated with CMS and CCAS, however, is still unclear. Damage to midline structures of the cerebellum, such as the fastigial nucleus, either by the tumor itself or by surgical intervention, has been discussed as the anatomical substrate [8,9].

The fastigial nucleus is located near the midline in the upper vermis immediately above the roof of the fourth ventricle and is therefore especially vulnerable during surgery for tumor resection involving cerebellar midline structures [10,11]. Besides its crucial role for axial motor function, there is growing evidence for its involvement in non-motor functions as it has anatomical connections to widespread areas, including the prefrontal cortex (PFC) via the ventral tegmental area and the mediodorsal thalamus [12,13,14]. Damage to this structure during tumor resection may therefore contribute to the development of CMS and CCAS [15,16].

Bilateral lesions of the fastigial nucleus in juvenile rats lead to impaired social interaction during development [15]. As adults, rats with fastigial lesions needed longer for learning a radial arm maze task and an auditory three-class oddball paradigm for attention and decision-making [16], which share some similarities to those of patients with CCAS. Nevertheless, learning the concept of a paradigm was primarily affected and, once the tasks were learned, performance was almost similar to that of controls. In anesthetized rats with fastigial lesions, electrophysiological recordings in the medial prefrontal cortex (mPFC) revealed compromised single unit activity, alongside enhanced local field coherence between the mPFC and somatosensory cortex across various frequency bands [16] together with epigenetic dysregulation of the glutamate transmitter system [17].

However, behavioral and electrophysiological investigations have been conducted separately. So far it therefore remains open whether there is a direct functional relationship between behavioral performance and mPFC neural activity. The auditory three-class oddball paradigm requires a behavioral action upon a rare target tone, while ignoring a rare distractor tone and a frequent standard tone of different frequencies. The term “oddball” refers to the rare deviant stimuli that stand out from a sequence of standard repetitive stimuli. This task design probes selective attention and stimulus evaluation, as it relies on detecting infrequent, behaviorally relevant stimuli among frequent, irrelevant ones.

During oddball paradigms, event-related potentials (ERPs) are analyzed. These are time-locked voltage fluctuations in the neural local field potentials (LFPs) that reflect sensory and cognitive processing stages following stimulus presentation. We recently showed that using this paradigm in rats allows us to derive ERPs with high similarities to recordings in human research [18]. Here we aimed to determine how compromised mPFC activity, resulting from fastigial nucleus lesions inflicted in juvenile rats, modulates neural processing of auditory stimuli in a three-class oddball paradigm.

## 2. Materials and Methods

### 2.1. Animal Husbandry

Male Sprague Dawley rats (*n* = 29, Charles River Laboratories, >200 g at start of training) were used for this study. The animals were housed in groups of 2–3 animals in a Makrolon Type IV cage within a scantainer (Scanbur, Karlslunde, Denmark) under a controlled environment (22 ± 2 °C; 55 ± 10% humidity; 10/14 h dark/light cycle with lights on at 06:00 a.m.) and with access to water at all times. Standard rodent chow (Altromin, Lage, Germany) was fed with 16 g per day and animal to enhance salience of reward pellets during behavioral testing while still ensuring weight gain until about 400–500 g body weight. Clinical scores and body weight were measured at least twice a week to ensure the well-being of the rats, in addition to daily visual inspections to monitor their health status. All efforts were undertaken to minimize the number of animals used and their suffering. Notably, recordings of the naïve control rats have also been used in our previously published manuscript [18]. This reuse of naïve control rats’ datasets is in line with ethical concerns by minimizing the need for new animal experiments and contributes to justifying animal experimentation.

### 2.2. Course of the Study

**Figure 1:**
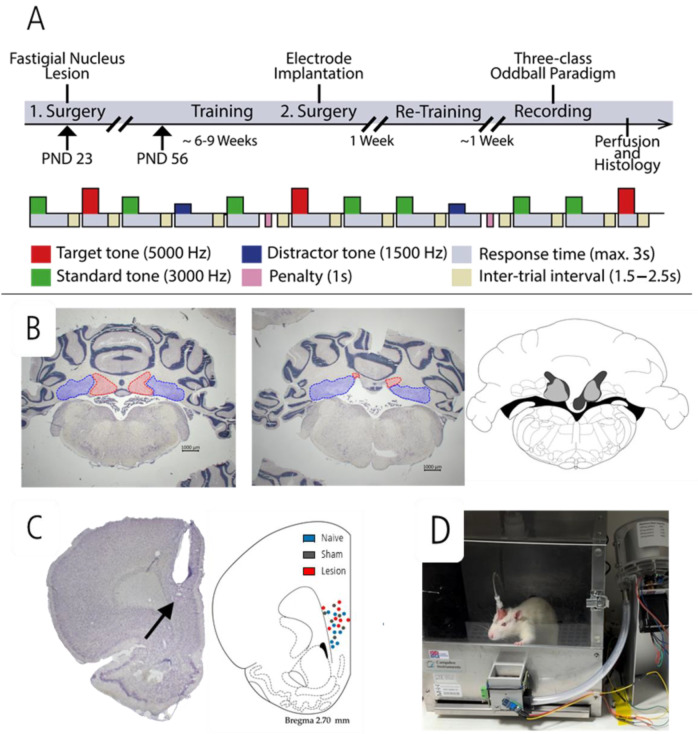
Schematic timeline of experimental study and the three-class auditory oddball paradigm (**A**). Illustration of the fastigial nucleus (red) in a sham-lesioned animal (**left**), along with histological evidence from a lesioned animal (**middle**) showing the absence of the fastigial nucleus. The interposed nucleus (blue) remains unaffected. The representative coronal section (**right**) illustrates the largest (dark gray) and smallest (light gray) lesion extents within the fastigial nucleus (**B**). Localization of the electrode in the prefrontal cortex of all groups (**C**). Behavioral box used for training and testing rats during cable-bound electrophysiological recordings (**D**).

At postnatal day (PND) 23, juvenile rats were assigned to three different groups: the lesion group received bilateral lesions of the fastigial nucleus (*n* = 12), rats in the sham-lesioned group underwent a sham surgical procedure that left the nucleus intact (*n* = 8), and unoperated naïve controls (*n* = 9). In early adulthood (PND 56), all rats were trained in the three-class oddball paradigm, after which electrodes were implanted in the mPFC for cable-bound electrophysiological recordings during behavioral testing. Neuronal recordings in the three-class oddball paradigm were conducted in a single session which lasted about 60 min. After completion of behavioral testing, the positions of the electrodes in the mPFC and the locations of the lesions were verified by histological examination of Nissl-stained brain sections (see timeline in Figure 1A).

### 2.3. Fastigial Nucleus Lesion

The stereotaxic surgery was performed under general chloral hydrate anesthesia (360 mg/kg) with local anesthesia (Lidocaine) and systemic analgesia (Carprofen: 5 mg/kg intraoperatively, 2.5 mg/kg postoperatively for two days). Rats were placed in a stereotaxic apparatus (Stoelting, Wood Dale, IL, USA) with atraumatic ear bars during surgery. All animals who underwent the surgery were lesioned at the following coordinates with reference to Lambda [19]: Anterior–Posterior: −2.5/−3.0 mm, Lateral: ±1 mm, Dorso-Ventral: −6.4 mm. To induce the lesion, 300 µA was applied for 60 s, which typically results in localized lesions of less than one cubic millimeter, as required for the fastigial nucleus [16]. Sham-lesioned animals underwent surgery, too, but the electrode was placed 2 mm above target and no current was applied.

### 2.4. Oddball Paradigm

Eight weeks after lesion, training in the three-class auditory oddball paradigm was started in rat 5/9-hole boxes (80600A Operant Chamber, Campden Instruments Limited, Loughborough, UK) with one open hole located on the back panel and a small container with a flap in the front panel for reward pellets (Dustless Precision Pellets 45 mg, Rodent Purified Diet, BioServ) provided by a food magazine. Infrared sensors detected nose pokes into the back hole. The next trial was initiated when the rat took the pellet out of the container. The stimulus schedules and the data acquisition were controlled by the Presentation^®^ software (Neurobehavioral Systems Inc., Berkeley, CA, USA), which also sent information to a microcontroller (Arduino) that controls the feeder’s stepper motor.

During the three-class oddball paradigm, the rats had to respond to a rare target tone (5000 Hz) by a nose poke while ignoring a frequent standard tone (3000 Hz), and an infrequent distractor tone (1500 Hz). Standard, target and distractor tones, all 100 ms long, were presented at a sound pressure level of about 80 dB in a ratio of 6:2:2. The response time window (trial duration) was 3000 ms. Between tone signals, an inter-trial interval was randomly set between 1500 and 2500 ms. Responding to a target tone led to a target “hit” count. Correct ignoring of a standard or distractor tone led to a correct rejection (CR) count. Figure 1A provides a schematic overview of the auditory three-class oddball paradigm.

For the three-class oddball paradigm, animals were trained for several weeks prior to stereotaxic implantation of the electrodes into the mPFC. Starting with only target tones at a duration of several seconds, standard and distractor tones were subsequently introduced. Training difficulty increased over time until it reached the criteria above, as described in [18] in more detail.

The following parameters were used to compare behavior between different groups: the number of days until the animals reached criterion of ≥70% correct behavior. In fully trained rats, the reaction time to nose pokes in the back hole after target was used as a measure for motor response. The number of nose pokes to each tone (target, distractor and standard) was counted and the ratio of correct behavior was calculated. In addition, the d-prime value (d’) was calculated as a measure for sensitivity using the hit rate for the target tone and the false alarm rate of the distractor tone, according to the following formula: d’ = Z(hit rate) − Z(false alarm rate).

### 2.5. Electrode Implantation

For this procedure, the same pain management and anesthesia regime was used as for the fastigial nucleus lesion. As additional treatment for prevention of infection at the headstage connector, antibiotic Marbofloxacin (Marbocyl^®^, Ismaning, Germany, 10 mg in 1 mL/kg) was given subcutaneously for eight days starting two days before surgery. The tooth bar was set at -3.3 mm during surgery. A handmade unipolar electrode of insulated platinum–iridium wire (90:10; 101R-3T; Science-Products GmbH, Hofheim am Taunus, Germany), coated with Teflon (uncoated ∅ 76.2 µm, coated ∅ 140 µm), was used. The wire was placed in a stainless-steel tube 13 mm long cut from a 24G syringe needle (outer ∅: 0.45 mm; inner ∅: 0.25 mm, Sterican Braun Melsungen AG, Melsungen, Germany), and an uncoated wire tip was exposed by 0.45–0.5 mm. All animals were implanted with an electrode into the right mPFC with the following coordinates in reference to Bregma [19]: Anterior–Posterior: −2.7 mm, Lateral: +0.8 mm, Dorso–Ventral: −4.4 mm from skull. A jeweler’s screw with attached wire was inserted near the recording electrode and used as reference. Additionally, a wire-attached jeweler’s screw inserted into the skull above the cerebellum served as the grounding electrode. All implants were attached with dental acrylic (Paladur^®^, Hanau, Germany) to anchor screws. A headstage connector was formed to allow recording of neuronal activity via a flexible cable in the awake, freely moving rat during the behavioral experiment. All rats were monitored on a warming mat until they were fully awakened. After surgery, the rats were kept in individual cages overnight and then returned to their cage mates. Following surgery, rats were given a one-week recovery period before resuming behavioral training.

### 2.6. Electrophysiology Recording

Cable-bound recordings of neural activity were completed during behavioral testing in the oddball paradigm and nose pokes in response to the target tone, as well as the correct rejection of the distractor and standard tones aligned with neural activity. For the neuronal recording, the rat headstage was connected with a cable to the electrode adaptor box (Figure 1D). A swivel was interposed to protect the cable from twisting and to allow free movement during behavioral testing in the operant chamber. LFP signals were amplified (isolated) 1000-fold and band-limited (band pass, 0.5–100 Hz) with an analogical amplifier (Signal Conditioner Cambridge 1902; Cambridge Electronic Design, Cambridge, UK). The signals were then digitized (Cambridge Electronic Design CED 1401 Mark II) with a resolution of 1000 Hz and saved for further computations (Spike II software v.6). From the digitized signals, 50 Hz frequency was removed with a notch filter to remove the electrical power interference. Neuronal signals were synchronized with auditory stimuli of the oddball paradigm as trigger events, while rat behavioral responses (nose poke) were tracked by the Presentation^®^ software (Neurobehavioral Systems Inc., Berkeley, CA, USA). All behavioral responses and event marker information were then sent to Spike2 with a specific code through the amplifier (CED 1401) and displayed in real time on the computer together with the brain activity.

### 2.7. Data Processing

All data processing steps were completed using a PC (Windows 10). For the acquisition of the ERP data, the software Python (version: 3.11) with the PyCharm community Editor (version: 2022.2.1) was used. For the extraction of data from Spike II to Python, the packages Neo (version: 0.12.0) and NumPy (version: 1.24.3) were used. At first, the data were distributed to their associated variables stored as NumPy arrays. Raw data were filtered offline with a lowpass filter of 30 Hz (as performed in [20]) and epoched from −200 ms to 800 ms around stimulus onset. First, the individual ERPs of the different groups to target, distractor and standard tone were visualized for each group to define our areas of interest for the early and the late components, ensuring that the peak amplitudes of all rats remained within the predefined areas. The early component is thought to reflect sensory encoding and stimulus differentiation, while the late component is associated with higher-order processes such as attention allocation and response preparation [18]. Afterwards, a grand average was calculated with our defined areas of interest. For the grand average data, all trials were treated equally by creating a mean. The data of the oddball paradigms were normalized into Z-score transformation (subtracting the mean and dividing by the standard deviation across the entire epoch). For the baseline correction, the mean voltage during the pre-stimulus interval (−200 to 0 ms) was subtracted; in addition, trials were rejected if the signal exceeded ±3 standard deviations from the mean within the baseline interval (−200 to 0 ms), indicating potential artifacts. Next, the peak amplitude and peak latencies of the early and late components of the grand average ERPs for correct behavior to target, distractor and standard were determined. Thereafter, the peak amplitudes and their latency within our area of interest were determined for each animal and its corresponding trials. Further methodological details regarding task design, trial structure and ERP validation have been published previously [18].

### 2.8. Histology

Rats were euthanized under deep anesthesia and underwent transcardial perfusion with phosphate-buffered saline (Sigma-Aldrich, St. Louis, MO, USA) followed by 4% paraformaldehyde solution. Afterwards, the brains were extracted and postfixed in 4% paraformaldehyde and 30% sucrose solution (1:1) for at least 24 h. Thereafter, they were transferred to pure 30% sucrose solution for an additional 24 h to ensure cryoprotection. Using a cryostat (Leica Microsystems, Wetzlar, Germany), coronal brain sections were cut at a thickness of 30 µm for the cerebellum and 40 µm for the cerebrum. For the cerebellum, four series were collected and every 120 µm a section was mounted onto Superfrost slides, covering the entire cerebellum. For the cerebrum, five series were collected, with one section mounted every 200 µm onto gelatin-coated slides. Standard Nissl staining with Thionin was performed to visualize cell bodies. Lesion extent and electrode placement were evaluated by visual inspection under a Zeiss light microscope by an experienced observer. Special attention was given to confirm bilateral fastigial damage and to ensure that the interposed nuclei remained unaffected. Electrode locations in the medial prefrontal cortex were identified based on gliosis and visible electrode tracks, using anatomical landmarks from a rat brain atlas [19].

### 2.9. Statistical Analysis

Statistical analysis was performed with SigmaPlot15 (Systat Software Inc., Palo Alto, CA, USA) and with GraphPad Prism 10 (GraphPad Software, Inc., 2023, Boston, MA, USA). For comparison between naïve, sham-lesioned and lesioned groups, the training days until criterion of ≥70% correct behavior, as well as the reaction times and correct behavior after target and the d’ value during electrophysiological recordings in the oddball paradigm, a one-way ANOVA with factor “group” was applied.

For the electrophysiological analysis of ERP differences, we first performed a mass-univariate one-way ANOVA at each time point within the time window from −200 ms to 800 ms post-stimulus, comparing the naïve, sham-lesioned and lesioned groups separately for target, distractor and standard stimuli. Post hoc comparisons were conducted using Welch’s *t*-test and corrected for multiple comparisons using the false discovery rate (*p* = 0.05). In addition, we analyzed the amplitude and peak latency of the early and late components of the ERPs with a two-way ANOVA used with factors “group” and “stimuli”. ANOVA was followed by post hoc comparison with the Tukey Test in case of significance (*p* < 0.05). All tests were performed two-sided and *p* < 0.05 was considered significant. Graphs were plotted using GraphPad Prism 10 (GraphPad Software, Inc., 2023, Boston, MA, USA). All ERP visualizations were made with Python using Matplotlib (version: 3.7.1).

## 3. Results

Three rats were sacrificed within 24 h after the first surgery because of bad health scores (one lesioned rat and two sham-lesioned rats). One more rat from the lesion group was sacrificed as it presented with sudden onset seizure activity four days after surgery. All other rats recovered from surgery, with initial mild ataxia and weight loss (<10%) seen in the lesioned group which recovered within the first week. Histological analysis showed no lesion of the fastigial nucleus in one rat. In all other rats, bilateral lesions of the fastigial nucleus were verified together with electrode tips located in the right mPFC (Figure 1B,C). Thus, nine naïve animals, six sham-lesioned animals and nine lesioned animals were used for statistical analysis.

### Behavior and ERPs of Neural Recordings

**Figure 2:**
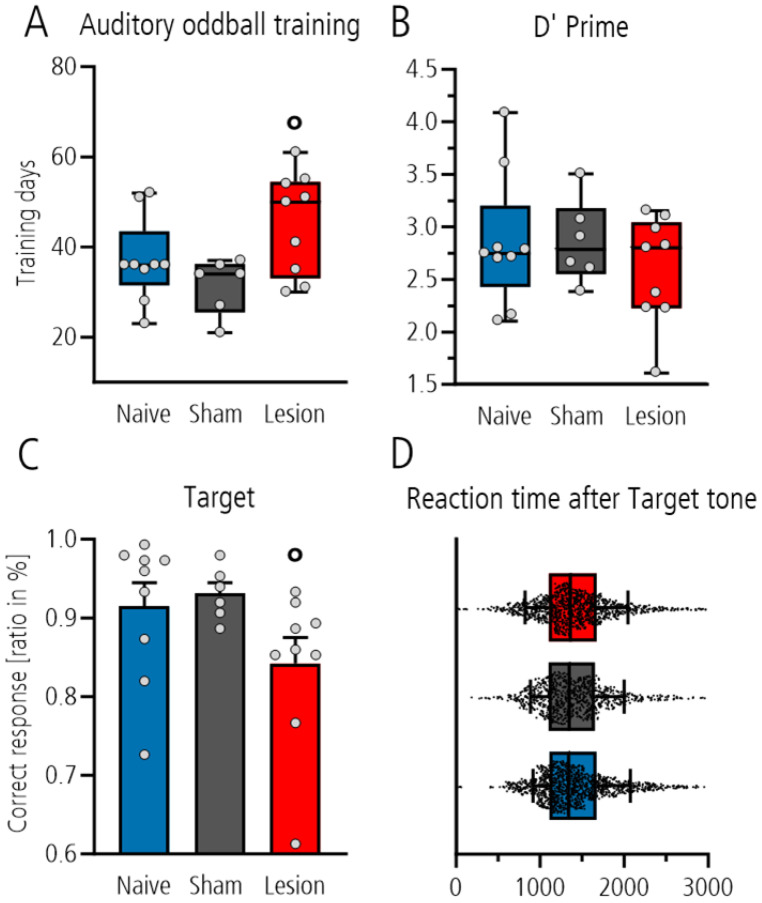
Graphs show the days until rats reached criterion of 70% correct behavior during training (**A**), as well as the d-prime (d’) value between target and distractor (**B**), the correct response ratio for target (**C**), and the reaction time of correct response to target (**D**) in fully trained rats during behavioral testing with cable-bound recording. Data are shown as box plot showing median with 10–90% percentile range (**A**,**B**,**D**), and bar graph with mean + S.E.M with individual data points superimposed (**C**) for naïve (blue), sham (gray) and lesion rats (red). Significant differences between sham and lesioned group are shown as circle (°; post hoc testing with *p* < 0.05).

We first compared the number of training days required to reach the criterion of ≥70% correct behavior in the three-class oddball paradigm in the different groups. Statistical analysis showed that the lesioned group needed more days on average than the sham-operated control group (shown by significant post hoc Tukey pairwise comparison between the sham-lesioned and lesioned group, *p* = 0.032, after one-way ANOVA with *p* = 0.034; Figure 2A), whereas the comparison to the naïve group did not reach the level of significance. In fully trained rats, behavioral analysis during electrophysiological recordings showed no difference between groups with respect to the d’ value as measure for the sensitivity to discriminate between the target and distractor tones (one-way ANOVA, *p* = 0.513, Figure 2B). Nevertheless, analysis of the ratio of correct responses to target tone showed that the lesioned group performed worse than the sham-lesioned or naïve controls (Mann–Whitney *t*-test *p* = 0.018 after one-way ANOVA with *p* = 0.10; Figure 2C). This was not a result of compromised motor activity as the reaction time for poking did not differ between groups (one-way ANOVA *p* = 0.3114; Figure 2D). In contrast, analysis of the ratio of correct rejection to the distractor and standard tones showed no group differences (one-way ANOVA: *p* = 0.422 and *p* = 0.394).

After baseline correction, the number of included trials was reduced to 1152 target hit trials, 1107 distractor CR trials and 4893 standard CR trials for the naïve group. For the sham-lesioned group, 784 target hit, 764 distractor CR and 3338 standard trials, and, for the lesioned group, 1078 target hit, 1195 distractor CR and 5240 standard CR trials were included. For statistical analysis, the number of standard trials was randomly reduced to match the respective number of target trials in each group, ensuring comparable group sizes and preventing biased statistical outcomes due to unequal trial counts.

**Figure 3:**
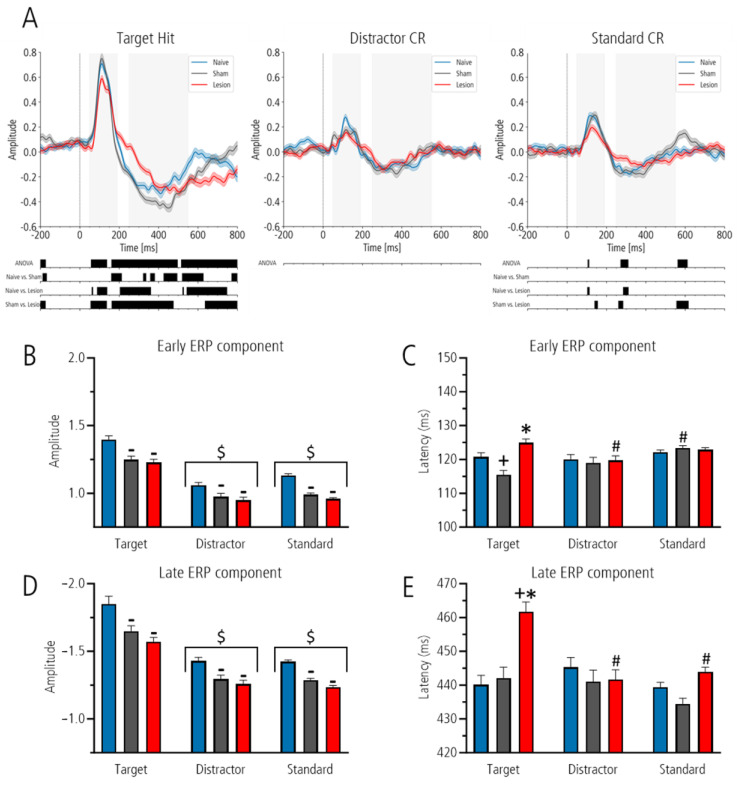
Event-related potentials (ERPs) derived during behavioral testing in an auditory three-class oddball paradigm with S.E.M and below one-way ANOVA following post hoc test. Black bars indicating significant areas (**A**). The naïve group (blue), sham-lesioned group (gray) and lesion group (red) are shown for the following conditions: target hit (left), correct rejection (CR) of distractor (middle) and standard (right). Shaded areas indicate specific time intervals of interest. Bar charts illustrate the early (**B**,**C**) and the late (**D**,**E**) components of the ERPs as bar graphs + S.E.M. with amplitude (**B**,**D**) and latency (**C**,**E**). Significant post hoc comparisons after significant ANOVA within the stimuli (target, distractor and standard) to the naïve group are marked with asterisks (+; *p* < 0.05), and to the sham group with circles (*; *p* < 0.05). Comparison within the groups (naïve, sham and lesion) are indicated with hashtags (#; *p* < 0.05) for differences relative to target and with plus signs (+; *p* < 0.05) for differences relative to distractor. Comparisons between whole stimuli relative to the target are marked with dollar signs ($; *p* < 0.05). Comparisons between factor “group” to naïve rats are marked with minus signs (-; *p* < 0.05).

Analysis with mass-univariate one-way ANOVA between ERPs of naïve, sham-lesioned and lesioned groups for target revealed three significant clusters: 57–138 ms, 161–497 ms and 515–800 ms (all *p*-values < 0.02; all F-values > 3.8). Post hoc testing with Welch’s test showed that within the 800 ms post-stimulus interval, 70% of time points after target was flattened in the lesioned compared to the sham-lesioned group and 53% compared to the naïve, whereas 37% differed between naïve and sham-lesioned animals (all *p* < 0.05; see Figure 3A for details). In the sham-lesioned group, a significant difference was also detected in the pre-stimulus interval of the target hit condition, which was not observed in other conditions.

While analysis after distractor stimuli showed no significant differences between ERPs, analysis after the standard stimulus highlighted three significant time clusters: 105–112 ms, 272–311 ms and 562–612 ms (all *p*-values < 0.029; all F-values > 5.8). Post hoc analyses revealed no significant differences between the naïve and sham-lesioned groups. However, significant differences were found between naïve and lesioned (5% of all time points) and between sham-lesioned and lesioned animals (13% of all time points), indicating a flatter curve of the lesioned group in these time intervals.

Second, for the analysis of ERP amplitudes and latencies, we defined time windows for peak detection based on visual inspection of the grand averages for target, distractor and standard ERP (Figure 3A). For the early component the area between 50 and 190 ms was defined, and for the late component the area between 250 and 550 ms was defined.

In all groups, the amplitude of the early and late components was highest for target as compared to distractor and standard. However, the amplitude of the early and late ERP components was lowest in the lesioned group as compared to sham-lesioned and naïve controls for all stimuli. Moreover, the peak latency after target was prolonged only in the lesioned group. For all conditions (amplitude and latency of the early and late ERP components), a two-way ANOVA with the factors “stimuli” and “group” revealed a significant effect for the factor “stimuli” (all F-values between 2.3 and 120, all *p*-values between 0.097 and 0.001) and the factor “group” (all F-values between 3.3 and 32.3, all *p*-values between 0.001 and 0.037). Interactions between factors were only found for the early and late ERP peak latencies (both F-values 4.3 and 6.6, with *p*-values 0.002 and 0.001), whereas for the early and late ERP amplitudes the interaction between factors were not significant.

Therefore, in the following, only results of post hoc group comparison is described: early and late peak amplitude of the ERPs. Comparison between groups showed that lesioned groups had the lowest amplitude, followed by that of the sham-lesioned and that of the naïve controls (all *p* < 0.01) but with no significance between sham-lesioned and lesioned group for the early component. Post hoc comparison between stimuli showed that, for the early and late components, the amplitude to the target was significantly higher as compared to standard and distractor (both *p* < 0.001; Figure 3B,D).

Early and late peak latency of the ERPs: for the early ERP component, the peak latency of the lesioned group after target tone was significantly prolonged as compared to that of the sham-lesioned group (*p* < 0.001), an effect that did not reach the level of significance compared to the naïve controls (*p* = 0.062). Groups did not differ with respect to peak latency after distractor and standard. In addition, in the lesioned group the peak latency after target was significantly longer as compared to that of distractor (*p* = 0.011). Also, the latency of the sham-lesioned group after target was reduced as compared to that after standard (*p* = 0.001).

For the late ERP component, the peak latency of the lesioned group after target was significantly longer than that of the sham-lesioned and naïve controls (*p* < 0.001). Groups did not differ with respect to latency after distractor and standard. In addition, the latency of the lesioned group after target was prolonged to that after distractor and standard (*p* < 0.001), whereas the latency of the sham-lesioned and the naïve control group did not differ between stimuli (Figure 3C,E).

## 4. Discussion

The cerebellum has not only been recognized as a structure involved in motor control, but also in higher-order cognitive functions [21]. More recently, it has even been described as a “coordinator” that modulates the activity of the cerebral cortex and thereby indirectly influences cognitive processes [22]. This would support the concept that cognitive and emotional impairments described in CCAS are associated with cerebellar damage [5,23]. We showed here that in rats of juvenile age with a lesion of the fastigial nucleus, target detection is reduced, which is accompanied by a remarkable flattened ERP response during testing in the three-class oddball paradigm. This pattern clearly suggests an influence of the fastigial nucleus on stimulus processing in the mPFC. Moreover, the proportion of correct responses to target tones was mildly, but consistently, reduced. These behavioral changes suggest impairments in attention and decision-making, which correspond to core domains affected in CCAS.

Efferent cerebellar pathways reach the cerebral cortex via deep midline nuclei, with the fastigial nucleus being the most medially located [10]. The fastigial nucleus is therefore particularly vulnerable to damage during neurosurgical procedures aimed at removing tumors in the midline vermis during childhood. As the fastigial nucleus serves as the primary relay for tracts originating from the vermian cortex and maintains polysynaptic connections to widespread brain regions, including the PFC [13,14], it is increasingly recognized for its role in cognitive and affective processing [22]. Supporting this, studies demonstrated that lesions of the fastigial nucleus in pediatric patients increase the risk for postoperative mutism [24,25,26].

Studies on long-term survivors after posterior fossa surgery in childhood report persistent deficits of social behavior and intellectual performance many years after surgery, corresponding to CCAS [27]. Correspondingly, rats of a juvenile age with bilateral lesions of the fastigial nucleus are cognitively impaired, which mainly presents as difficulties in acquiring the concept of behavioral paradigms, resulting in significantly more training days to learn the three-class oddball paradigm. However, once the paradigm was learned, all groups showed comparable discrimination ability for target and distractor tones; only correct responding to the target tone was marginally disturbed in the lesioned group. This aligns with previous studies in our group, where we showed that bilateral lesions of the fastigial nucleus in juvenile rats result in impairments to learn the concept of the complex radial-maze task and the three-class oddball paradigm during training in adulthood [16]. While our findings indicate disturbed attention and learning in lesioned animals, which are core features of CCAS, the present data do not allow a conclusive assessment of other cognitive domains affected in the syndrome. Therefore, the link between fastigial lesions, altered mPFC activity and CCAS-like impairments should be interpreted within the scope of the tested functions.

Analysis of ERPs elicited by specific stimuli during behavioral testing in oddball paradigms have been widely used to investigate the neural mechanisms underlying cognitive functions and information processing [28]. Early ERP components are associated with simple stimulus processing and primarily driven by the physical properties of a stimulus, such as brightness in visual paradigms [29], whereas late ERP components are considered markers of cognitive processing and are associated with stimulus evaluation and attentional allocation [28]. In humans, the late ERP component is represented by the so-called P300, as it typically occurs around 300 ms after stimulus onset in the PFC [28,30], whereas in rats the P3-like late ERP component is observed approximately between 200 and 500 ms after target stimulus onset [31].

In the present study, more detailed analysis of mPFC ERPs during behavioral testing in the three-class oddball paradigm showed that the peak amplitude of the early and late ERP components is reduced in rats with fastigial nucleus lesions as compared to sham-lesioned or naïve controls. In humans, a reduced P300 ERP amplitude is typically associated with lower engagement of attentional and cognitive processing resources, although attenuation of the P300 amplitude has also been reported in the context of increased task difficulty, where greater cognitive effort is directed toward task execution. In such cases, reduced amplitude may reflect increased mental workload rather than diminished cognitive capacity per se [32,33,34]. Although behavioral performance seemed largely intact, the altered ERPs suggest that the neural mechanism involved in task performance differed in lesioned animals.

We also showed that, in rats with fastigial lesions, the peak latency of the late ERP component after the target tone is prolonged, although the rats’ behavioral reaction times to target tones do not differ from those of control groups. In the human context, the P300 peak latency is considered an index of the time required for stimulus evaluation and decision-making [34,35,36], and prolonged P300 latencies after target stimuli are interpreted as markers of slowed cognitive processing or increased mental effort. Interestingly, in patients with cerebellar lesions, prolonged ERP latencies have been described, which have been linked to cognitive impairments and which further support the translational parallel between our animal model and human pathology [37]. Also, in autism spectrum disorder (ASD), which has a strong neurodevelopmental background, abnormalities in the P300 component have been related to deficits in cognitive processing [38,39]. A reduction in P300 amplitude in ASD has been interpreted as a generalized impairment in stimulus evaluation resulting from attentional and cognitive dysfunction and unnecessary over-detailed information processing [38], while prolonged latency of the late P300 component in response to novel stimuli suggests that individuals with ASD may over-process information necessary for discriminating between target and novel stimuli. Interestingly, in individuals with ASD hypoplasia of vermal lobules VI and VII, regions with strong connections to the fastigial nucleus are among the most frequently reported cerebellar abnormalities [40].

Together, the reduced peak amplitude, combined with prolonged peak latency of the late ERP component seen in our rat model with juvenile induced fastigial lesions, are interesting in the human context. Alterations in late ERP components have been linked to mild cognitive impairment, episodic memory deficits and dysfunctions in attention and working memory (summarized in [37]). In this context, the amplitude of late P300 ERPs is thought to reflect the amount of cognitive resources allocated during stimulus evaluation, while their peak latency is considered as an index of the processing time required before generating a behavioral response [41]. Consistent with this, patients with cerebellar lesions show a prolonged latency and reduced amplitude of the P300 component [37].

It is noteworthy that, although rats with fastigial nucleus lesions show impairments in acquiring complex learning and attention paradigms, their task performance, once the task is learned, only marginally differs from that of sham-lesioned or naïve controls [16]. The marked ERP abnormalities in the PFC during behavioral testing in the oddball paradigm may thus reflect compensatory or adaptive neural reorganization, enabling behavioral adaptation despite underlying neural dysfunction. Such compensations may involve increased recruitment of alternative neural pathways that connect cerebellar regions with the PFC after fastigial lesions. These findings, however, highlight the importance of ERP measures in assessing the long-term cognitive consequences of cerebellar lesions.

Future studies should also address broader cognitive and affective roles of the fastigial nucleus, as previous studies also showed disturbed radial maze learning and enhanced anxiety in the elevated plus maze [16]. Furthermore, adult lesion groups should be included to determine whether the observed deficits are unique to early-life disruption or generalize across the lifespan.

Taken together, our results suggest that rats with fastigial lesions require increased cognitive effort to process auditory stimuli, likely due to lesion-induced disturbance of communication between the cerebellum and prefrontal cortex. The prolonged ERP latency in response to target tones may especially reflect a slowed decision-making process, a characteristic commonly observed in individuals with cerebellar damage [37].

A limitation of the present study is that, although less pronounced, the amplitudes of the early and late ERP components of sham-lesioned animals were also reduced as compared to naïve controls despite preservation of the fastigial nucleus. Similar observations have been reported in previous studies, where sham-lesioned groups also displayed functional alterations despite the absence of structural damage of the fastigial nucleus [15,16]. These effects may be attributed to a small lesion or mild gliosis caused by the transient insertion of the electrodes, e.g., via the cerebellar vermis, which provides most afferent fibers to the fastigial nuclei [16,42]. Furthermore, the difference between the sham-lesion versus the naive and the lesioned groups in the pre-stimulus interval may reflect anticipatory processes such as arousal, attention or motor preparation [43,44,45,46]. However, the observed difference lacked consistency across comparisons and did not relate to stimulus processing per se; this may also be due to the fact that ERP baselines were normalized. One further issue may be that visual inspection of electrode depth showed that, in lesioned rats, the electrodes were placed in more dorsal parts of the mPFC, while the electrodes of sham-lesioned and naïve controls were placed in dorsal and ventral parts, which may have affected the outcome of ERP measures. However, analysis of ERPs derived by dorsal and ventral placed electrodes did not differ. Finally, so far our lesion model allows assessment of persistent effects in adulthood but does not address functional reversibility. Future studies may therefore apply reversible approaches, such as pharmacological inhibition or cerebellar stimulation, to test whether behavioral and ERP alterations can be modulated. This would help distinguish compensatory from non-compensatory effects and clarify the functional relevance of the observed deficits.

## 5. Conclusions

This study provides compelling evidence that lesions of the fastigial nucleus in juvenile rats lead to lasting neurophysiological impairments, characterized by reduced ERP amplitudes and delayed latencies of adult rats tested in an auditory oddball paradigm. These findings highlight the cerebellum’s crucial role in attention, decision-making and sensory discrimination, likely mediated by its extensive connectivity with prefrontal cortical areas. For future studies, our translational animal model will enable a detailed analysis of the underlying neurobiological mechanisms, which will give additional insight into correlational clinical studies addressing CMS and CCAS. Ultimately, these insights may support the development of targeted neurosurgical strategies and therapeutic interventions that further preserve cognitive function in pediatric patients with midline cerebellar tumors.

## Data Availability

All data and materials are presented in the manuscript, and additional supporting files are available from the corresponding author upon reasonable request.

## Abbreviations

The following abbreviations are used in this manuscript:

ASD	Autism spectrum disorder
CCAS	Cerebellar cognitive affective syndrome
CMS	Cerebellar mutism syndrome
CR	Correct rejection
d′	d-prime
ERP	Event-related potential
LFP	Local field potential
mPFC	Medial prefrontal cortex
PFC	Prefrontal cortex
PND	Postnatal day
